# Automatic brain tissue segmentation based on graph filter

**DOI:** 10.1186/s12880-018-0252-x

**Published:** 2018-05-09

**Authors:** Youyong Kong, Xiaopeng Chen, Jiasong Wu, Pinzheng Zhang, Yang Chen, Huazhong Shu

**Affiliations:** 1 Laboratory of Image Science and Technology, Key Laboratory of Computer Network and Information Integration, School of Computer Science and Engineering, Southeast University, Nanjing, People’s Republic of China; 2 International Joint Laboratory of Information Display and Visualization, Nanjing, People’s Republic of China

**Keywords:** Magnetic resonance imaging, Brain tissue segmentation, Supervoxel generation, Graph filter

## Abstract

**Background:**

Accurate segmentation of brain tissues from magnetic resonance imaging (MRI) is of significant importance in clinical applications and neuroscience research. Accurate segmentation is challenging due to the tissue heterogeneity, which is caused by noise, bias filed and partial volume effects.

**Methods:**

To overcome this limitation, this paper presents a novel algorithm for brain tissue segmentation based on supervoxel and graph filter. Firstly, an effective supervoxel method is employed to generate effective supervoxels for the 3D MRI image. Secondly, the supervoxels are classified into different types of tissues based on filtering of graph signals.

**Results:**

The performance is evaluated on the BrainWeb 18 dataset and the Internet Brain Segmentation Repository (IBSR) 18 dataset. The proposed method achieves mean dice similarity coefficient (DSC) of 0.94, 0.92 and 0.90 for the segmentation of white matter (WM), grey matter (GM) and cerebrospinal fluid (CSF) for BrainWeb 18 dataset, and mean DSC of 0.85, 0.87 and 0.57 for the segmentation of WM, GM and CSF for IBSR18 dataset.

**Conclusions:**

The proposed approach can well discriminate different types of brain tissues from the brain MRI image, which has high potential to be applied for clinical applications.

## Background

Magnetic resonance imaging (MRI) has been widely employed to examine the anatomical structures of the human brain in both clinical application and neuroscience research [1, 2]. Compared to other medical imaging modalities, MRI has the advantage of the high spatial resolution and well soft-tissue contrasts [3, 4]. This powerful technique can yield exquisite differentiation between different types of tissues, including white matter (WM), grey matter (GM) and cerebrospinal fluid (CSF). Accurate segmentation of these tissues is of significant importance in the several applications [5–7]. Manual segmentation is extremely time-consuming due to millions of voxels in the brain MRI image. Besides, the segmentation result is prone to substantial intra-observer and inter-observer variation. Therefore, it is essential to propose an effective approach for automatic and accurate segmentation of brain tissues from the MRI image.

Accurate segmentation can be challenging due to the tissue heterogeneity, which is caused by noise, bias filed and partial volume effects in brain MRI [8, 9]. To overcome these issues, great deals of efforts have been made to propose a number of approaches for brain tissue segmentation in the past two decades. These methods can be mainly categorized into three main-streams, i.e. level set methods [10], classification approaches [11–13] and atlas based methods [14]. The level set methods in natural images are extent to the brain tissue segmentation, which are sensitive to user initialization and parameter settings [10]. Several clustering methods have been employed for brain tissue segmentation, such as the Gaussian mixture model [11] and fuzzy C-means [12]. Besides, the tissue atlases have been employed to propose effective segmentation methods to enable accurate segmentation of tissues [14].

The existing voxel-vise segmentation approach for MRI have drawbacks in neglecting the spatial information among data. Fortunately, the promising supervoxel technique provides a possible solution to make use of the statistical information of the local regions. In the past decade, this technique has been increasingly employed for natural image processing and analysis in the fields of computer vision and machine learning [15–17]. This powerful technique can group similar voxels into a meaningful supervoxel. The brain MRI image consists of approximately piecewise constant regions, which is suitable for generating appropriate supervoxels. Therefore, the supervoxel technique has been recently utilized for several brain MRI analysis applications, such as segmentation, registration and functional parcellation [18–21]. However, with approach supervoxels, it is still challenging to enable robust segmentation due to the tissue heterogeneity [22, 23].

To cope with these issues, this paper proposes an effective algorithm for the brain tissue segmentation based on supervoxel and graph filter. A novel distance metric is proposed to develop an efficient and effective supervoxel generation method to suppress the noise in the MRI image. After computing features from each supervoxel, a graph filter algorithm is employed to classify these supervoxels into different types of brain tissues. Experiments on two widely utilized MRI dataset demonstrate the superior performance of the proposed approach compared to the state-of-art voxel-vise and supervoxel based brain MRI segmentation algorithms. The proposed method achieves mean dice similarity coefficient (DSC) of 0.94, 0.92 and 0.90 for the segmentation of white matter (WM), grey matter (GM) and cerebrospinal fluid (CSF) for BrainWeb 18 dataset, and mean DSC of 0.85, 0.87 and 0.57 for the segmentation of WM, GM and CSF for IBSR18 dataset.

## Methods

### Materials

The MRI datasets in this study includes the BrainWeb 18 MRI dataset [24] and the Internet Brain Segmentation Repository (IBSR) dataset [25]. The BrainWeb 18 dataset has 18 MRI images from the McConnell Brain Imaging Centre. The images are simulated with different level of noise ranging from 0 to 9% and with an intensity non-uniformity (INU) level of 0, 20% or 40%. Each image includes 181 × 217 × 60 voxels with 1 mm × 1 mm × 1 mm. The IBSR dataset is consisting of 18 real MRI images derived from healthy subjects. Each MRI volume has a size of 256 × 256 × 128 voxels with 1 mm × 1 mm × 2 mm. All the images in the two datasets are provided with the ground truth tissue segmentation of WM, GM and CSF.

### Methods

The proposed method mainly includes two steps, i.e. supervoxel generation and supervoxel classification. An effective supervoxel algorithm is first employed to over segment the brain MRI volume into a number of small compact supervoxels with homogenous appearance. These supervoxels are then classified into different types of tissues based on graph filter.

#### Supervoxel generation for brain MRI

In the past decade, a number of algorithms have been proposed to generate meaningful supervoxels with homogeneous regions. The commonly used algorithms are normalized cuts, mean shift, turbo pixels and the simple linear iterative clustering (SLIC) method. Normalized cuts [26] recursively partitions a graph to globally minimize the cost function. Its high computational complexity limits its application for images with a large size. Mean shift [27] is a gradient based method, which generates supervoxels by recursively moving to the kernel smoothed centroid. This approach cannot control the size and the compactness of supervoxels, which may produce irregular supervoxels. The turbo pixel method [28] perform supervoxel generation by evolving the geometric flow from seeds sampled uniformly on the image plane. The SLIC method [29] employs the k-means clustering to classify neighborhood voxels into each seed for generating compact supervoxels. This efficient method can control the number and compactness of the supervoxels.

Among these methods, the SLIC method has been widely applied for the high dimensional MRI images due to its efficiency [30–32]. SLIC initially generates a number of cluster centers sampled at regular intervals of length at each dimension of the image plane or volume. The length *L* is calculated by the number of voxel *N* and the number of supervoxels *q* as $L=\sqrt[3]{N/q}$. The cluster centers are then perturbed to the lowest position in a neighborhood to avoid placing them at an edge. The voxel within a 2*L* × 2*L* × 2*L* area round the center on the xyz plane are clustered into each supervoxel based on their intensity and location similarity, which guarantees the homogeneity and compactness of supervoxels. After clustering all the voxels into the nearest cluster center, a new center is calculated as the average intensity and spatial positions of all the voxels belonging to this cluster. The process of clustering is iteratively repeated until that the distance between the new centers and the previous ones is smaller than a threshold.

In the SLIC algorithm, images are considered as approximately uniform for homogeneous regions. However, MRI images are always contaminated by the Rician noise in the imaging process. The voxel similarity measurements in the SLIC method may be unreliable on MRI images. To overcome this problem, a novel voxel similarity measurement is proposed to suppress the noise as

1 $$ {\displaystyle \begin{array}{c}{d}_{int}=\left\Vert {G}^{\ast }{I}_{N_i}-{I}_c\right\Vert \\ {}d={d}_{int}+\gamma {d}_{\mathrm{spa}}\end{array}} $$

where ${I}_{N_i}$ represent the intensity matrices of the cubic image patches with central voxel *N*_*i*_ and *I*_*c*_denotes the intensity of seed *c*. Function *G* represents the standard Gaussian kernel and ∗ denotes the convolution operator. The intensity distance between the voxel *N*_*i*_ and the seed *c* is denoted as *d*_*int*_, and the spatial similarity between the voxels and seed is represented as *d*_*spa*_. A regularization parameter *γ* is introduced to weigh the relative importance between voxel intensity and the spatial proximity. The regularization parameter is set 0.2 empirically in this study. The parameter of Gaussian kernel is adopted to the noise level estimated using the median absolute deviation method [33].

**Fig. 1:**
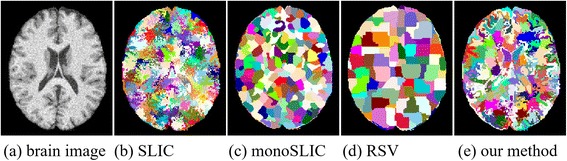
Supervoxels of a noisy brain MRI image using SLIC and our proposed method. **a** is the original brain MRI image, **b**, **c**, **d** and **e** are the supervoxel results from the SLIC, monoSLIC, RSV and the proposed method

Figure 1 illustrates the supervoxels generated from a noisy brain MRI image using the SLIC, monoSLIC [34], regularity preserved supervoxel (RSV) method [35] and our proposed method. It can be easily seen that the original SLIC method is sensitive to the noise, and generate irregular supervoxels. MonoSLIC and RSV methods can generate regular supervoxels with a bad adherence of the tissue boundaries, especially at the cortex regions. Our approach can guarantee the boundaries of supervoxels to adhere well the brain tissue boundaries in image. Furthermore, our suepervoxel algorithm makes the size of supervoxel as regular as possible. The proposed supervoxel technique has high potential to be applied for the MRI images of other organs or other medical images, such as computed tomography and ultrasound imaging [36, 37].

#### Supervoxel classification based on graph filter

After generating supervoxels for the 3D brain MRI image, it is essential to develop an effective approach to classify these supervoxels into different types of tissues to enable an accurate segmentation. Here we propose to classify these supervoxels based on an effective graph filter approach [38]. An undirected weighted graph G = {V,A} is constructed for the MRI image. As there are huge number of voxels in the brain MRI volume, we construct a graph among the above generated supervoxels, which can highly reduce the computational complexity. The nodes V = {*v*_1_, *v*_2_, …, *v*_*N*_} of the graph are supervoxels, and A is a N × N weighted adjacency matrix. Each node is represented by the intensity features derived from the corresponding supervoxel. The weight between each node is obtained by computing the similarity between the nodes using the radial basis function between the features

2 $$ \mathrm{A}\left(i,j\right)=\exp \left\{-\frac{{\left({v}_i-{v}_j\right)}^2}{2\alpha}\right\} $$

where v_i_ represents the value of feature in node i and *α* represents hyper parameter. As the supervoxels in the same class have similar features, each node is only connected to K nearest neighbors in the graph.

Similar to traditional digital signal processing, filters can be performed on the graph signal. It has been demonstrated that a graph filter H can be linear and shift invariant with the assumption of the equation between the characteristic and minimal polynomials of the adjacency matrix. The graph filter is defined as

3 $$ \mathrm{H}=\mathrm{h}\left(\mathrm{A}\right)={\sum}_{l=0}^L{h}_l{A}^l $$

where A is the adjacency matrix, and L represents the taps of filter.

This study proposes a semi-supervised segmentation approach with a few number of known labels. The segmentation is performed by classification of supervoxels using an adaptive graph filter. The graph filter propagates the nodes of known labels to predict the nodes of unknown labels, defined as

4 $$ {s}^{predict}=\mathrm{h}\left(\mathrm{A}\right){s}^{known} $$

The optimal taps can be adaptively determined by adaptively constructing filter based on the initial labels. For the nodes *V*^*known*^ of the the initial known labels, the the label *s*^*known*^ is set to − 1 or 1 and the unknown labels are set to 0. A smaller subset of training nodes *V*^*train*^ is selected from the nodes with known labels *V*^*known*^. A graph filter can be found from the training nodes that correctly classify the nodes in *V*^*known*^. An adaptive filter can thus be estimated from the selected training nodes with a least square minimization problem

5 $$ argmin\ \left\Vert Dh(A)\ {s}^{train}-{1}_N\right\Vert $$

where *D* =  *diag* (*s*^*known*^) is the diagonal matrix with initial known labels on its main diagonal. The binary classifier can be extent to the multi-class classification in brain tissue segmentation by the one-against-all strategy.

## Results

The performance of the proposed method was evaluated on two widely used datasets, including the BrainWeb 18 MRI dataset [24] and the Internet Brain Segmentation Repository (IBSR) dataset [25]. The number of supervoxel was set to 4000 empirically for these two datasets in the experiments and the taps of filter L was set to 4 in the experiments.

The performance of the proposed approach is compared with other state-of-the-arts, including two voxel-wise methods and one promising supervoxel based methods. The two voxel-wise methods are the FMRIB Software Library v5.0 (FSL) [39] and Statistical Parametric Mapping (SPM8) [40], which are widely used in the neuroscience community. The FSL software employs expectation maximization algorithm with hidden Markov random field model for segmentation. The SPM8 tool utilizes atlas based approach with the probabilistic atlases of brain tissues. The supervoxel based method is a recently developed approach especially for brain tissue segmentation [20].

**Fig. 2:**
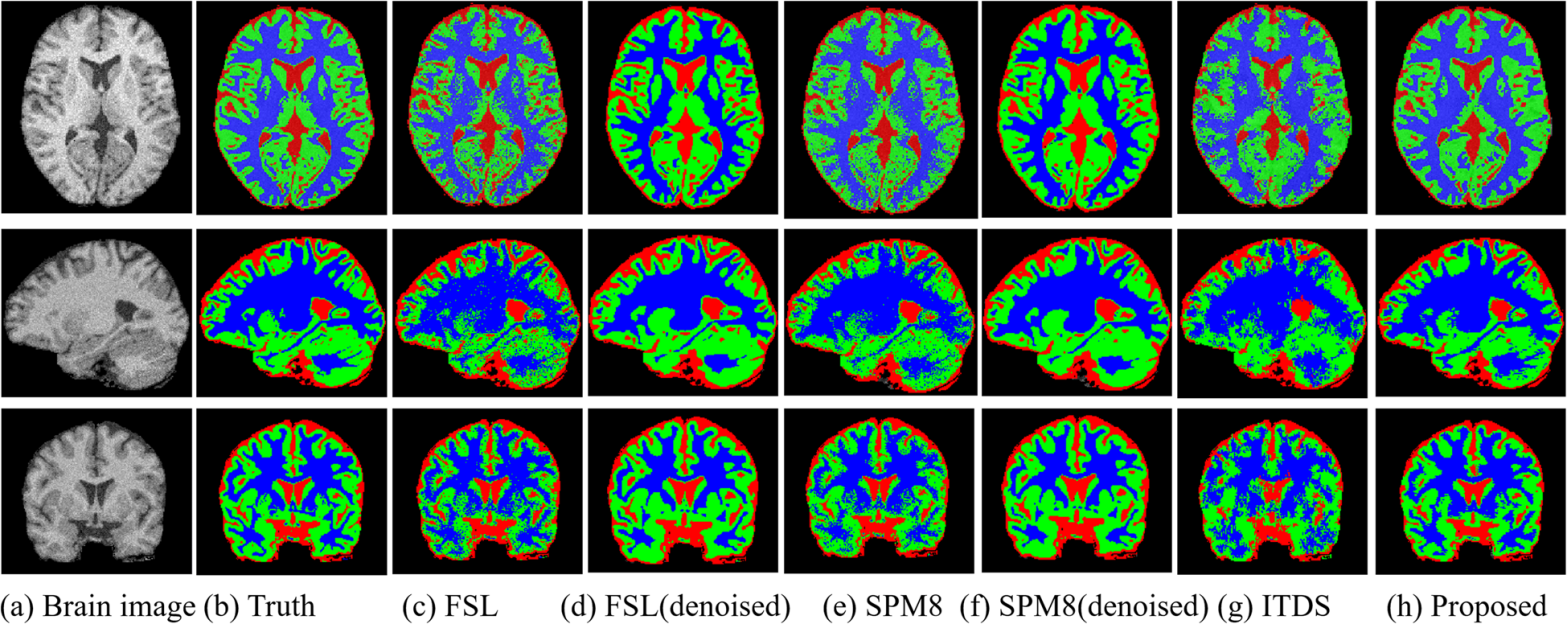
Segmentation results using different methods for the brain image with 9% noise and INU level of 40% from the BrainWeb18 dataset. The colors of red, green and blue represents the labels of segmentation for cerebrospinal fluid (CSF), grey matter (GM) and white matter (WM). The three rows are the two dimensional axial, sagittal and coronal views of three dimensional segmentation results, respectively. Column (**a**) and (**b**) are the original image and the ground truth of the segmentation. Column (**c**-**g**) are the segmentation results of the FSL, FSL on denoised data, SPM8, SPM8 on denoised data, ITDS and the proposed method

Figure 2 illustrates the brain tissue segmentation results of the image volume with a noise level of 9% and an INU level of 40% from the BrainWeb dataset. In the figure, the 2D axial, sagittal and coronal views of 3D segmentation results are shown for visual inspections using the itk-snap tool [41]. The first and second columns are the image and the ground truth of the segmentation with red, green, blue voxels corresponding to CSF, GM and WM tissues, respectively. The third, fourth, fifth, sixth, seventh and eighth column illustrate the segmentation results of the FSL, FSL on denoised data, SPM8, SPM8 on denoised data, ITDS methods and our proposed algorithm. As the ground truth for reference, we can observe the advantage of our proposed segmentation over other three approaches. In particular, the proposed method shows apparently better delineations of the WM and GM tissues. With the results on the denoised data with FSL (the fifth column) and SPM8(the seventh column), the FSL and SPM method cannot obtain better results on denoised MRI volumes [33]. This may be because that these two segmentation models consider the noise in the MRI volume, and the denoised data corrupt the intrinsic features.

To further assess the performance objectively, quantitative evaluation is performed by calculating the dice similarity coefficient (DSC) and volume difference ratio (VDR) [42–44]. These metrics are commonly utilized benchmark evaluation strategies in the segmentation community. The DSC metric measures the similarity between automatic segmentation results and ground truth for each tissue, defined as

6 $$ DSC=\frac{2\times \mathrm{TP}}{2\times \mathrm{TP}+\mathrm{FP}+\mathrm{FN}} $$

The VDR metric measures the volume difference between and the ground truth and the achieved segmentation results, defined a s

7 $$ VDR=\frac{\left|\mathrm{FP}-\mathrm{FN}\right|}{\mathrm{TP}+\mathrm{FN}} $$

where TP, FP and FN are the numbers of true positive, false positive and false negative voxels, respectively. A higher value of DSC and a lower value of VDR represent a better correspondence to the ground truth, which denotes a higher accuracy of the segmentation results.

Tables 1 and 2 shows the performance of the segmentation results for each method on the BrainWeb18 and IBSR18 datasets, respectively. As for the BrainWeb18 dataset, the proposed method provides the highest DSC values on CSF, GM and WM, followed by SPM8, FSL and ITDS. The ITDS method using traditional SLIC supervoxel method is sensitive to the noise in the images from the BrainWeb18 dataset. As for the IBSR18 dataset, the proposed method achieves best performance in segmentation of CSF and GM, and obtains comparable accuracy of WM segmentation compared with the other three methods. The ITDS can achieve better performance than the SPM8 and FSL for the low level of noise in the images of the IBSR18 dataset. The VDR value is quite lower than other three methods for the two datasets and especially for the IBSR18 dataset. This is because that the proposed method under-estimate the CSF tissue due to the small training number of supervoxels.

**Table 1:** Performance of segmentation results on the BrainWeb18 dataset

	DSC			VDR		
	WM	GM	CSF	WM	GM	CSF
FSL	0.91 ± 0.04	0.88 ± 0.03	0.89 ± 0.01	0.10 ± 0.08	0.08 ± 0.05	0.24 ± 0.01
SPM8	0.91 ± 0.03	0.90 ± 0.02	0.89 ± 0.03	0.06 ± 0.05	0.05 ± 0.01	0.14 ± 0.06
ITDS	0.90 ± 0.05	0.88 ± 0.05	0.88 ± 0.04	0.07 ± 0.03	0.06 ± 0.02	0.12 ± 0.05
Proposed	0.94 ± 0.01	0.92 ± 0.01	0.90 ± 0.01	0.02 ± 0.01	0.02 ± 0.01	0.04 ± 0.01

**Table 2:** Performance of segmentation results on the IBSR18 dataset

	DSC			VDR		
	WM	GM	CSF	WM	GM	CSF
FSL	0.87 ± 0.03	0.76 ± 0.03	0.53 ± 0.06	0.11 ± 0.13	0.23 ± 0.04	1.32 ± 0.37
SPM8	0.87 ± 0.01	0.80 ± 0.04	0.55 ± 0.06	0.06 ± 0.04	0.18 ± 0.04	1.11 ± 0.39
ITDS	0.86 ± 0.02	0.81 ± 0.03	0.60 ± 0.05	0.07 ± 0.06	0.16 ± 0.05	0.75 ± 0.38
Proposed	0.85 ± 0.01	0.87 ± 0.03	0.57 ± 0.08	0.08 ± 0.05	0.04 ± 0.03	0.17 ± 0.10

**Fig. 3:**
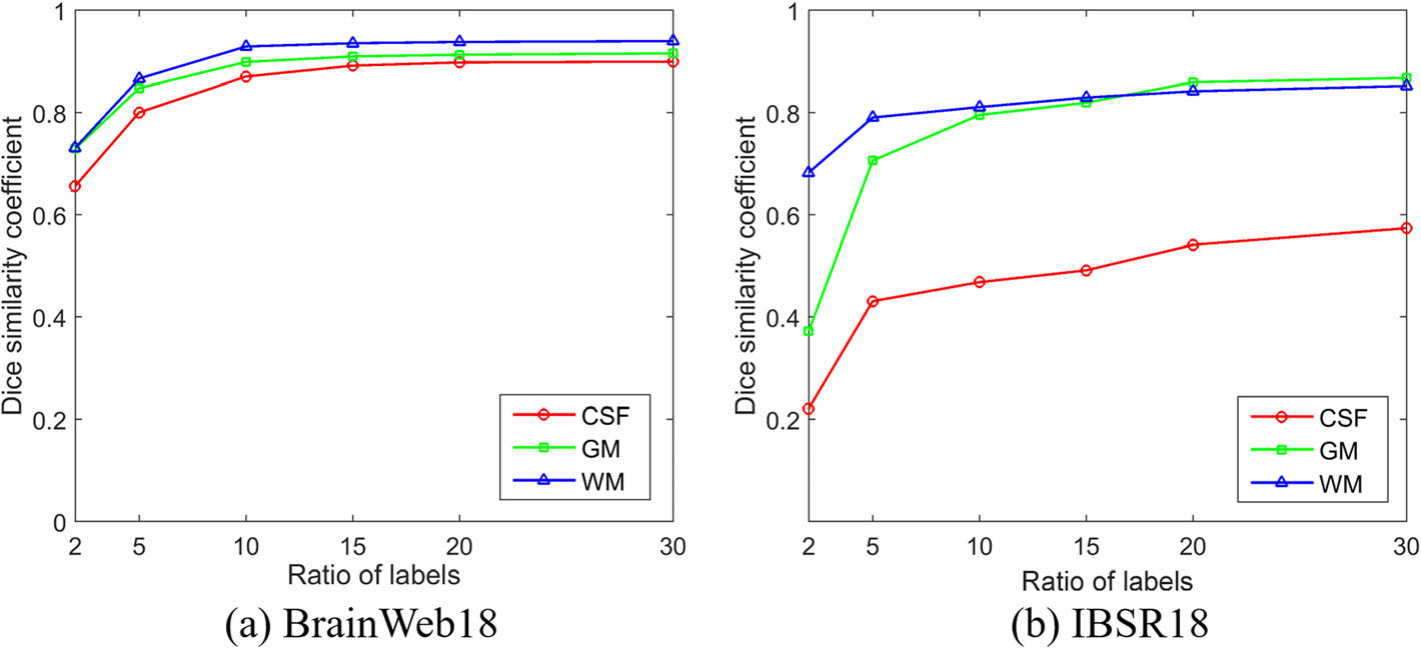
Dice similarity coefficients of segmentation results for the BrainWeb 18 and IBSR 18 datasets using our proposed method with different ratio of labels

**Fig. 4:**
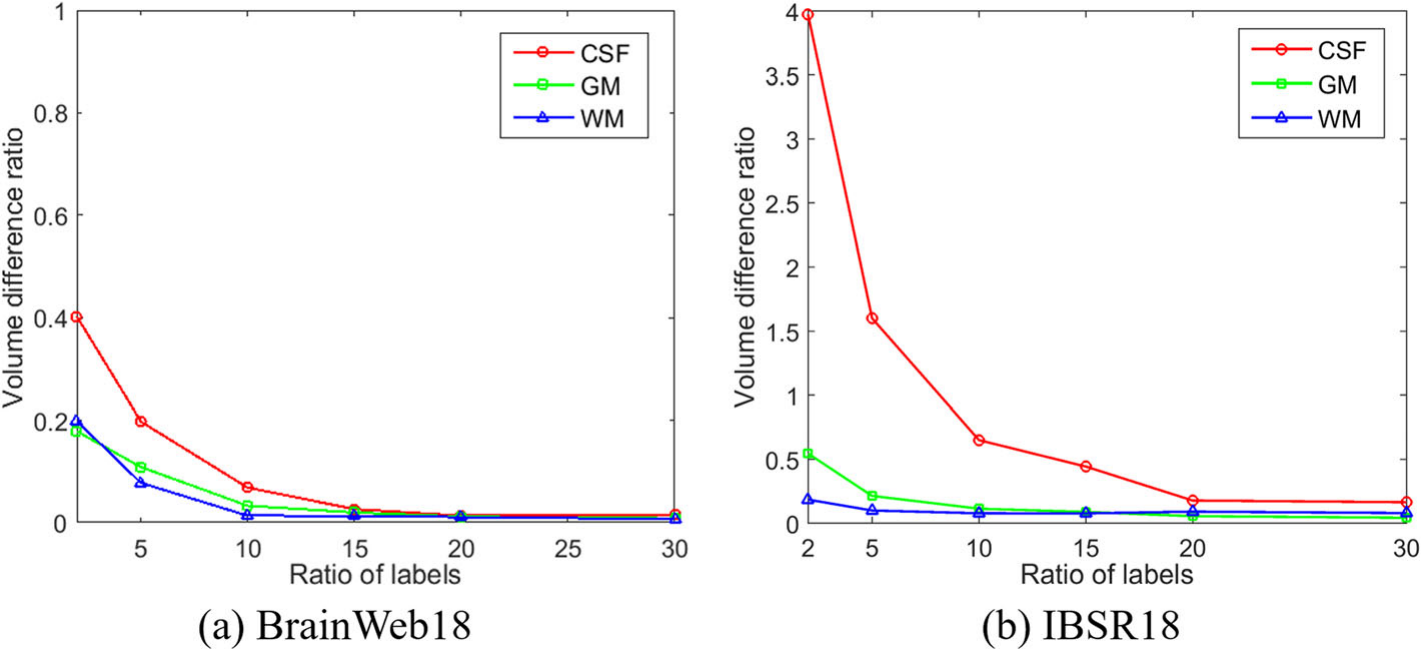
Volume difference ratios of segmentation results for the BrainWeb 18 and IBSR 18 datasets using our proposed method with different ratio of labels

TIn the above experiments, the ratio of labels is set to 0.3 for each brain MRI image. We further investigate the effect of the ratios for the segmentation accuracy in the proposed framework. The segmentation accuracies for each type of tissue are computed at the ratio of 0.02, 0.05, 0.1, 0.15, 0.2 and 0.3 for both the BrainWeb18 and IBSR18 dataset. Figures 3 and 4 illustrates the average DSC and VDR values of different volumes for the tissues of WM, GM and CSF at different ratio of known labels.

To further evaluate the effectiveness of the proposed supervoxel method, we compare the performance with the monSLIC and RSV method. All the methods generate about 4000 supervoxels, and same parameters are set with 30% labels with each types of tissues in the classification stage. For the BrainWeb datasets, the DSC values of results with the monSLIC method are 0.74, 0.71, 0.60 for WM, GM and CSF, and DSC values of results with the RSV method are 0.70, 0.72, and 0.69. For the IBSR datasets, the DSC values of results with the monSLIC method are 0.78, 0.84 and 0.53 for WM, GM and CSF, and DSC values of results with the RSV method are 0.67, 0.78 and 0.40. Our method obtains better performance than other two popular supervoxels for the two datasets.

## Discussion

The results on the two commonly utilized datasets demonstrated the effectiveness of the proposed brain tissue segmentation algorithm based on both qualitative and quantitative evaluations. The performance was achieved due to the appropriate supervoxels and the effective clustering approach. At first, a novel supervoxel method was developed to suppress the influence of noise in MRI to guarantee the boundaries of supervoxels to adhere well the brain tissue boundaries in image. Secondly, an effective semi-supervised clustering algorithm was proposed based on graph filtering to classify the supervoxels into three types of tissues.

For the performance on the two datasets, there were dramatically decrease accuracy of CSF in the IBSR 18 compared with those from the BrainWeb18 dataset. There are two main reasons for such results. Firstly, the BrainWeb18 dataset is a simulated dataset, and the IBSR18 dataset is a real dataset. The BrainWeb 18 dataset can be easy segmented while it is more difficult for the IBSR18 dataset. Secondly, among the three tissues, CSF has a small number of voxels compared to GM and WM. The employed DSC and VDR values measure the overlapping ratio and volume difference between automatic segmentation results and ground truth. Therefore, a same number of wrong segmented voxels can lead to a larger decrease of the DSC values or a large increase of VDR values for CSF compared with the other two tissues.

For the supervoxel clustering, it is apparent that with a small ratio of labels, there was a relatively low segmentation performance for all three tissues. Increasing the ratio of labels can obtain consistent increasing segmentation accuracy of all the three tissues. There was a slight improvement of performance when the ratio of labels is more than 0.15, which was sufficient enough to learn the intrinsic features of different types of tissues.

## Conclusions

In this paper, we have proposed an effective algorithm for brain tissue segmentation from the MRI image based on supervoxel and graph filter. An effective supervoxel algorithm was proposed to suppress the noise influence in the MRI images. The supervoxels were then classified into three types of tissues integrating features using graph filter. Qualitative and quantitative evaluations on two widely utilized MRI datasets demonstrate the superior performance of the proposed approach compared to the state-of-art brain MRI segmentation algorithms.

## Abbreviations

CSF: Cerebrospinal fluid
DSC: Dice similarity coefficient
FSL: FMRIB Software Library
GM: Grey matter
IBSR: Internet Brain Segmentation Repository
INU: Intensity non-uniformity
MRI: Magnetic resonance imaging
RSV: Regularity preserved supervoxel
SLIC: Simple linear iterative clustering
SPM: Statistical Parametric Mapping
WM: White matter

## References

[1] Yang G, Nawaz T, Barrick TR, Howe FA, Slabaugh G (2015). Discrete wavelet transform-based whole-spectral and subspectral analysis for improved brain tumor clustering using single voxel MR spectroscopy. IEEE Trans Biomed Eng.

[2] Zheng H, Qu XB, Bai ZJ, Liu YS, Guo D, Dong JY, et al. Multi-contrast brain magnetic resonance image super-resolution using the local weight similarity. BMC Med Imaging. 2017;17(6):1–13. 10.1186/s12880-016-0176-2 PMC5240324 28095792

[3] Wang SH, Zhang Y, Zhan TM, Phillips P, Zhang YD, Liu G (2016). Pathological brain detection by artificial intelligence in magnetic resonance imaging scanning. Prog Electromagn Res.

[4] Sauwen N, Acou M, Sima DM, Veraart J, Maes F, Himmelreich U, et al. Semi-automated brain tumor segmentation on multi-parametric MRI using regularized non-negative matrix factorization. BMC Med Imaging. 2017;17(29):1–14. 10.1186/s12880-017-0198-4 PMC5418702 28472943

[5] Kong Y, Shi L, Hui SC, Wang D, Deng M, Chu WC (2014). Variation in anisotropy and diffusivity along the medulla oblongata and the whole spinal cord in adolescent idiopathic scoliosis: a pilot study using diffusion tensor imaging. AJNR Am J Neuroradiol.

[6] Magnoni S, Mac Donald CL, Esparza TJ, Conte V, Sorrell J, Macri M (2015). Quantitative assessments of traumatic axonal injury in human brain: concordance of microdialysis and advanced MRI. Brain.

[7] Deng Y, Ren ZQ, Kong YY, Bao F, Dai QH (2017). A hierarchical fused fuzzy deep neural network for data classification. IEEE Trans Fuzzy Syst.

[8] Wang S, Phillips P, Yang J, Sun P, Zhang Y (2016). Magnetic resonance brain classification by a novel binary particle swarm optimization with mutation and time-varying acceleration coefficients. Biomed Tech (Berl).

[9] Wang S, Zhang Y, Liu G, Phillips P, Yuan TF (2016). Detection of Alzheimer's disease by three-dimensional displacement field estimation in structural magnetic resonance imaging. J Alzheimers Dis.

[10] Li C, Huang R, Ding Z, Gatenby JC, Metaxas DN, Gore JC (2011). A level set method for image segmentation in the presence of intensity inhomogeneities with application to MRI. IEEE Trans Image Process.

[11] Greenspan H, Ruf A, Goldberger J (2006). Constrained Gaussian mixture model framework for automatic segmentation of MR brain images. IEEE Trans Med Imaging.

[12] Yang X, Fei B (2011). A multiscale and multiblock fuzzy C-means classification method for brain MR images. Med Phys.

[13] Song Z, Tustison N, Avants B, Gee J. Adaptive graph cutrs with tissue priors for brain MRI segmentation. IEEE Intern Sympo Biomed Imaging. 2006:762–5.

[14] Artaechevarria X, Munoz-Barrutia A, Ortiz-de-Solorzano C (2009). Combination strategies in multi-atlas image segmentation: application to brain MR data. IEEE Trans Med Imaging.

[15] Fan Y, Huchuan L, Ming-Hsuan Y (2014). Robust superpixel tracking. IEEE Transact Image Process.

[16] Deng Y, Bao F, Deng XS, Wang RP, Kong YY, Dai QH (2016). Deep and structured robust information theoretic learning for image analysis. Ieee T Image Process.

[17] Li ST, Lu T, Fang LY, Jia XP, Benediktsson JA (2016). Probabilistic fusion of pixel-level and Superpixel-level hyperspectral image classification. Ieee T Geosci Remote.

[18] Mahapatra D, Schueffler P, Tielbeek J, Makanyanga J, Stoker J, Taylor S, et al. Automatic detection and segmentation of Crohn's disease tissues from abdominal MRI. IEEE Trans Med Imaging. 2013;32(12):2332-47. 10.1109/TMI.2013.2282124 24058021

[19] Wu W, Chen AY, Zhao L, Corso JJ (2014). Brain tumor detection and segmentation in a CRF (conditional random fields) framework with pixel-pairwise affinity and superpixel-level features. Int J Comput Assist Radiol Surg.

[20] Kong Y, Deng Y, Dai Q (2015). Discriminative clustering and feature selection for brain MRI segmentation. IEEE Signal Processing Letters.

[21] Soltaninejad M, Yang G, Lambrou T, Allinson N, Jones TL, Barrick TR (2017). Automated brain tumour detection and segmentation using superpixel-based extremely randomized trees in FLAIR MRI. Int J Comput Assist Radiol Surg.

[22] Amoroso N, Errico R, Bruno S, Chincarini A, Garuccio E, Sensi F (2015). Hippocampal unified multi-atlas network (HUMAN): protocol and scale validation of a novel segmentation tool. Phys Med Biol.

[23] Morra JH, Tu Z, Apostolova LG, Green AE, Avedissian C, Madsen SK (2008). Validation of a fully automated 3D hippocampal segmentation method using subjects with Alzheimer's disease mild cognitive impairment, and elderly controls. NeuroImage.

[24] Kwan RK, Evans AC, Pike GB (1999). MRI simulation-based evaluation of image-processing and classification methods. IEEE Trans Med Imaging.

[25] Cocosco C, Kollokian V, Kwan R, Pike G, Evans A (1997). Brainweb: online interface to a 3D MRI simulated brain database. NeuroImage.

[26] Shi J, Malik J (2000). Normalized cuts and image segmentation. IEEE Trans Pattern Anal Mach Intell.

[27] Comaniciu D, Meer P (2002). Mean shift: a robust approach toward feature space analysis. IEEE Trans Pattern Anal Mach Intell.

[28] Lehmann F (2011). Turbo segmentation of textured images. IEEE Trans Pattern Anal Mach Intell.

[29] Achanta R, Shaji A, Smith K, Lucchi A, Fua P, Süsstrunk S (2012). SLIC Superpixels compared to state-of-the-art Superpixel methods. IEEE Trans Pattern Anal Mach Intell.

[30] Heinrich MP, Simpson IJ, Papiez BW, Brady SM, Schnabel JA (2016). Deformable image registration by combining uncertainty estimates from supervoxel belief propagation. Med Image Anal.

[31] Tian Z, Liu L, Zhang Z, Fei B (2016). Superpixel-based segmentation for 3D prostate MR images. IEEE Trans Med Imaging.

[32] Verma N, Cowperthwaite MC, Markey MK. Superpixels in brain MR image analysis. Conf Proc IEEE Eng Med Biol Soc. 2013;2013:1077-1080. 10.1109/EMBC.2013.6609691 24109878

[33] Coupe P, Manjon JV, Gedamu E, Arnold D, Robles M, Collins DL (2010). Robust Rician noise estimation for MR images. Med Image Anal.

[34] Holzer M, Rene D. Over-segmentation of 3D medical image volumes based on monogenic cues. Computer vision winter workshop. 2014. p. 35–42.

[35] Fu HZ, Cao XC, Tang D, Han YH, Xu D (2014). Regularity preserved Superpixels and Supervoxels. IEEE Transact Multimedia.

[36] Chen Y, Shi LY, Feng QJ, Yang J, Shu HZ, Luo LM (2014). Artifact suppressed dictionary learning for low-dose CT image processing. IEEE Trans Med Imaging.

[37] Chen Y, Budde A, Li K, Li YS, Hsieh J, Chen GH (2017). A platform-independent method to reduce CT truncation artifacts using discriminative dictionary representations. Med Phys.

[38] Sandryhaila A, Moura JMF. Discrete signal processing on graphs: graph filters. IEEE International Conference on Acoustics, Speech and Signal Processing. 2013. 6163–6.

[39] Jenkinson M, Beckmann CF, Behrens TE, Woolrich MW, Smith SM (2012). FSL. NeuroImage.

[40] Ashburner J, Friston KJ (2005). Unified segmentation. NeuroImage.

[41] Yushkevich PA, Piven J, Hazlett HC, Smith RG, Ho S, Gee JC (2006). User-guided 3D active contour segmentation of anatomical structures: significantly improved efficiency and reliability. NeuroImage.

[42] Kong Y, Wang D, Shi L, Hui SC, Chu WC (2014). Adaptive distance metric learning for diffusion tensor image segmentation. PLoS One.

[43] Deng Y, Kong YY, Bao F, Dai QH (2015). Sparse coding-inspired optimal trading system for HFT industry. Ieee T Ind Inform.

[44] Chen Y, Zhang YD, Yang J, Cao Q, Yang GY, Chen J (2016). Curve-like structure extraction using minimal path propagation with backtracking. Ieee T Image Process..

